# Hyperthermia-Induced Febrile Seizures Have Moderate and Transient Effects on Spatial Learning in Immature Rats

**DOI:** 10.1155/2015/924303

**Published:** 2015-04-30

**Authors:** Nawel Yagoubi, Yosra Jomni, Mohsen Sakly

**Affiliations:** Faculté des Sciences de Bizerte, Laboratoire de Physiologie Intégrée, Zarzouna, 7021 Bizerte, Tunisia

## Abstract

The aim of this study was to characterize a novel animal model hyperthermia-induced febrile seizure and to investigate the impacts of repetitive febrile seizures on spatial learning and memory performances in immature rats. *Methods*. Rats were subjected to hyperthermia exposure one, two, or three times in 10-day intervals during 30 min in a water bath warmed at 45–50°C and their behaviour was monitored. Morris water maze spatial learning and memory were examined for control and treated groups. Results showed that rats subjected to 30-minute hyperthermia hot water developed rapidly myoclonic jerks and then generalized seizures. After a single hyperthermia exposure, the time for generalised tonic-clonic seizures appearance was 16.08 ± 0.60 min and it decreased gradually with repetitive exposure to reach 12.46 ± 0.39 min by the third exposure. Febrile seizures altered the spatial learning and memory abilities in Morris water maze and increased the time spent to attain the platform after one or two exposures, while after a third exposure rats exhibited the same latency compared to controls. Similar results were obtained in probe test where rats, subjected to hyperthermia for one or two episodes, spent less time in the target quadrant compared to corresponding controls. Further, when platform was moved from northwest to southwest quadrant, memory transfer test indicated that after one or two hyperthermia exposures cognitive performances were slightly altered, while after a third exposure the latency to escape increased significantly compared to untreated group. It was concluded that 30 min of hyperthermia hot water was sufficient to induce febrile seizures in immature rats and an increase of susceptibility was observed with repetitive hyperthermia exposure. Hyperthermia treatment impaired cognitive performances but the effects were mostly transient and moderate.

## 1. Introduction

Epilepsy is a chronic neurological disorder which affects about 1% of the world population and which is more common during childhood than at any other age [1]. It is characterized by recurrent, spontaneous, and unexpected seizures which involve a loss of control. The intensity of seizures varies, ranging from mild blanks to full seizures or to “tonic-clonic seizures.”

Epilepsy in infants has the particularity of occurring in a developing brain. This could be the origin of possible cognitive-behavioral consequences [2]. Seizures and associated disorders generate learning disabilities and can sometimes lead to school failure [3].

These particular cognitive deficits are due to the location of a brain epileptogenic zone and to the mode of propagation of the electrical discharge. The neurons responsible for the electrical discharge are found in the temporal lobe, in the hippocampus more specifically. In some patients, the origins of this disorder are brain injuries, but in most cases there is no obvious organic cause. Temporal lobe epilepsy (TLE) is the most common and the most severe form of epilepsy in adults. It results in the loss of contact with reality and affects the memory. In a lithium-pilocarpine rat model of TLE, Ye et al. [4] provided experimental evidences that hippocampal myelinated fibers were degenerated with significantly less myelin basic protein expression and a decrease in the total volumes of hippocampal formations in comparison to control group.

Epilepsy appears in infants with a more heterogeneous semiology [5, 6]. However, children with this type of epilepsy exhibit worse memory performances than healthy children or those with other types of seizures [7, 8].

Most patients that underwent surgery for temporal lobe refractory epilepsy have suffered a prolonged febrile seizure during childhood [9]. A pathophysiological link between febrile seizures and TLE has never been established either in humans or in animal models [10, 11].

Fever is the most common symptom observed in the infant pathology [12]. In fact, febrile seizures are the most prevalent seizures in infants and children [13]. They occur in over 5% of children aged between 3 months and 6 years. Although febrile seizures are largely benign, complex febrile seizures increase the risk to develop TLE [14]. However, controversy still exists in terms of later cognitive incidence of febrile seizures [15].

Several animal models have been developed to investigate the pathogenesis of febrile seizures and their consequences [16, 17]. In the present study, we have characterised a novel immature rat model of hyperthermia-induced febrile seizures. Using this model we have investigated the effect of isolated or repetitive febrile seizures on spatial memory performances. Cognitive abilities were evaluated by using the Morris water maze test which is well characterized cognitive task of learning and memory.

## 2. Methods

### 2.1. Inducing Febrile Seizures

Neonates from pregnant Wistar rats were obtained from an inbred stain (Faculty of Sciences, Bizerta). The day of birth was considered as day 0 of postnatal life. When weaned (on P21), rats were housed 6 per cage and were kept at 25°C, under a 12:12 light/dark cycle with free access to food and water. Animals were cared of under the Tunisian code of practice for the care and use of animals for scientific purposes. The experimental protocols were approved by the Faculty Ethics Committee.

On postnatal day 11 (P11) [18], rats were introduced in individual glass bottles, covered with a perforated lid in order to provide breathing air. The bottles were placed in a water bath warmed at 45–50°C. Animals were kept in this hyperthermia situation for 30 min (core temperature > 39.5°C). Their behaviour was monitored by individual direct observation.

Following a trigger of myoclonic jerks, the rat was then transferred to the surface at room temperature. A few seconds later, the seizure turned into a tonic-clonic generalised seizure with vigorous shaking of the head, ears, and upper and lower limbs and an especially violent vibration of the tail. Most animals showed a reaction to the myoclonic febrile seizures and developed tonic-clonic generalised seizure.

A 30 min observation was then necessary to monitor the postictal state of the animals, which required less than 20–25 min to return to normal.

Each animal was rapidly cooled down with a few drops of water and dried out with paper towel in order to help it reach its natural body temperature. It then gradually resumed its activity and was returned to the cage.

Rats were exposed to one, two, or three hyperthermia episodes in 10-day intervals at postnatal days 11, 21, and 31 (P11, P21, and P31). Control rats were exposed to the same experimental conditions, excluding hyperthermia. These rats did not suffer any seizures, only moderate agitation due to the new environment.

### 2.2. Memory Test

Animals' spatial memory was tested with the Morris water maze [19]. This test assesses the animal's ability to store and manage spatial information in an aversive situation [20].

Treated and control rats underwent a spatial test after 2 days' rest in order to evaluate the impacts of febrile seizures on the spatial memory in the Morris water maze [21].

**Figure 1:**
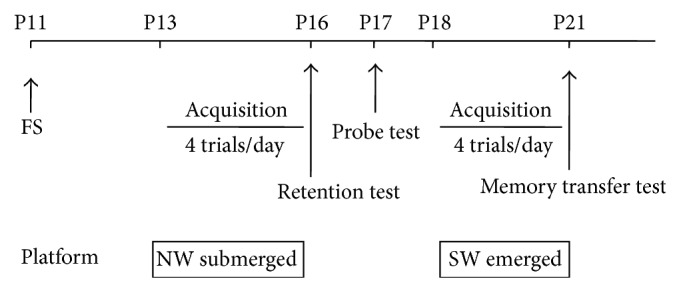
Treatment and behavioural testing protocol for treated group 1 (Tr1). The same procedure was applied for treated 2 and 3 groups. P11: postnatal day 11; FS: febrile seizure; NW: northwest; SW: southwest.

The test was carried out using a round pool (90 cm in diameter and 50 cm high) filled to a depth of 25 cm with water (22°C) and placed in the center of a room surrounded by several cues. The experiment was divided into various phases carried out in the following order (Figure 1). On the days 13–16, the rats were pretrained in order to learn the mechanism of the experiment (acquisition phase). Animals underwent four trials each day in the pool at four peripheral starting points (north, east, south, and west) in pseudorandom order and with an intertrial interval of 15 min. For each trial, the rat was gently placed in the water and allowed to swim for 60 s. When the rat did not manage to find the platform during 60 s, it was placed manually there by the experimenter for 10 s.

During pretraining the platform (20 cm in diameter) was located in a fixed position of the pool (middle of the northwest quadrant) and was made invisible to the rat by placing it 2 cm below the water surface covered by small polystyrene pieces.

On day 4 of the pretraining session and 20 min after the last trial, a retention test was performed. Latency to find the submerged platform was measured. Because a similar decrease of latency was recorded from the four starting positions, only north and west points were subsequently carried. Twenty-four hours after the retention test, a probe test was conducted. Rat was placed in the middle of the pool and the time spent in the quadrant that the platform was previously located in was recorded.

Twenty-four hours after the probe test, a second trial acquisition session was given under the same experimental conditions as the first test but with a visible platform lifted 1 cm above water level and placed in the southwest quadrant. On day 4, escape latency was measured after north or west introduction for each rat.

### 2.3. Statistical Analysis

The ANOVA parametric test was used, complemented by Tukey's test as a post hoc test to study the significance of differences between groups. Data are given as mean ± SEM values. *P* values < 0.05 were significant.

## 3. Results

### 3.1. Hyperthermia Treatment

As explained in the methods section, neonates (P11) were subjected to hyperthermia until they suffered a generalised tonic-clonic seizure (GTCS). In order to reach this stage, the rats had previously undergone myoclonic jerks. The time for myoclonic jerks to first appear (MJAT), the time for a GTCS to appear (GTCSAT), and the back to normal recovery time (RT) were recorded.

**Figure 2:**
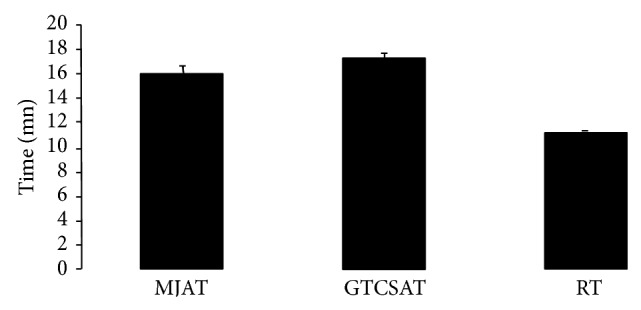
Myoclonic jerks appearance time (MJAT), generalised tonic-clonic seizure appearance time (GTCSAT), and recovery time (RT) after a first exposure (*n* = 15 for each group). Data are shown as mean values ± SEM.

Figure 2 showed that after a single exposure to hyperthermia, the GTCS was always triggered between a few seconds and one minute after the myoclonic jerks. The average MJAT was of the order of 16.90 ± 0.31 min, followed by the GTCSAT (17.98 ± 0.80 min), while the RT took 10.78 ± 0.31 min. These results indicate that our experimental model is efficient in inducing and tracking febrile seizure from myoclonic jerks until the GTCS in immature rats.

To examine the effect of repetitive hyperthermia exposure on time appearance of seizures, animals treated by a first exposure (Tr1) were exposed in an interval of 10 days a second (Tr2) and a third time (Tr3) to hyperthermia. In Tr3 group, the GTCS took less time to appear than in those exposed only once or twice to hyperthermia. The average GTCSAT for this group was 12.46 ± 0.39 min, while in Tr1 and Tr2 groups the GTCS appeared significantly later (*P* < 0.05) (Table 1).

The decrease of rat body weight before and immediately after seizures averaged 2.37, 2.20, and 1.94%, respectively, in Tr1, Tr2, and Tr3 groups. This decrease which was not significantly different compared to that of control rats (2.32, 1.96, and 1.14%, resp., in C1, C2, and C3) may be explained by fecal and urinary losses during confinement conditions.

### 3.2. Morris Water Maze

To examine the effect of repetitive febrile seizures on spatial learning and memory, we evaluated control and hyperthermia-treated rats for their performance in the Morris water maze which is employed in experiments as a basic assay of spatial memory and associated learning ability [21, 22].

Our results indicated that, by the fourth day of the acquisition phase of the task, Tr1 and Tr2 groups displayed more time than the corresponding controls to reach the submerged platform after a north or west introduction, suggesting that, after one or two hyperthermia exposures, rats did not improve their performance during the 4 days of pretraining (Table 2). However, after a third hyperthermia episode, rats exhibited the same latency compared to control groups to reach the goal following both north and west starting positions, indicating a possible functional and structural recuperation in Tr3 group. Furthermore, no significant differences were observed in escape latencies after west or north introduction.

During the probe test, both control and treated groups spent more time in the quadrant where the platform was located than would be predicted by chance (>15 min). However, treated rats Tr1 and Tr2 spent less significant time than corresponding controls in the target quadrant, while Tr3 group spent an equivalent time in this quadrant as C3 group. Nevertheless, in both hyperthermia-exposed and nonexposed groups, the quadrant dwelling time increased gradually with age (Table 3).

To check for the ability to learn a new location of the platform, we conducted a memory transfer test in which the platform was moved to the southwest quadrant and lifted 1 cm above water surface. Our results indicated that treated groups required generally more time to change the spatial reference of the previous environment to attain the emerged platform than corresponding controls, but the difference was significant only for Tr3 group after both north and west introductions as shown by the important increase of the latency to escape (Table 4).

## 4. Discussion

Febrile seizures occurred commonly in 2–5% of children under the age of 5 years and can be simple or complex [23, 24]. Complex febrile seizures early in life had a high risk for the later development of epilepsy and cognitive deficits [15].

In rats, as well as in humans, susceptibility to febrile convulsions is age-related. The highest sensitivity is found in young animals and it sharply decreases with age [25]. Several animal models have been developed to study the pathogenesis of febrile seizures and their consequences on cognitive functions. Baram et al. [26] had characterised a rat model of hyperthermia-induced seizures at P11 where brain maturity corresponded to the period of maximal sensibility to febrile seizures. In this model, the animals were introduced in a Plexiglas box with hot air (45–50°C) circulating inside it, powered by a hair-dryer which is placed under the box to progressively increase the body and brain temperature. In the present work, we have modified this model to prevent the animal draught and auditory stress from the hair-dryer.

Using this novel model, we showed that 30 min of hyperthermia hot water was sufficient to induce febrile seizures in P11, P21, and P31 rats. All hyperthermia-treated rats developed rapidly generalised seizures. This result is consistent with previous data suggesting that, in the immature healthy brain, a high and prolonged temperature is required for convulsive seizures to appear [27].

In accord with a previous study [13], we recorded a decrease of rat body weight of about 2% before and immediately after seizures similar to that of untreated rats. This decrease which did not result in dehydratation, since clinical dehydratation in children was defined by 5% body weight loss [28], may be mainly related to urinary and fecal losses.

In rats subjected to hyperthermia for two or three episodes in 10-day intervals, the generalised seizure appearance time was reduced by 18 and 23%, respectively, indicating an enhancement of seizure susceptibility with repetitive hyperthermia exposure, probably due to thermoregulatory centres maturation.

Retrospective studies have shown that febrile seizures induced damage in the medial temporal structures and thus led to hippocampal sclerosis and to TLE [29, 30]. It has also been demonstrated that, overall, generalised tonic-clonic seizures and complex partial seizures had the greatest negative effects on cognitive abilities [31].

Our research was directed to check the impact of single or repetitive prolonged febrile seizures on memory function in immature rats. To this end, we have used the Morris water maze which is mainly a hippocampus-dependent spatial learning and memory task [19, 32].

By the fourth learning day, only rats of control group C3 improved their performance to attain the aim after north or west introduction. In contrast, Tr1 and Tr2 groups displayed significantly more time to reach the submerged platform suggesting that after one or two hyperthermia exposures, rats did not improve their performance during the 4 days of pretraining and solely Tr3 group displayed the same ability as the corresponding control after north or west introduction. It may be concluded that, by the third episode of hyperthermia, rats acquired the task similarly as untreated groups. Indeed, hyperthermia-induced seizures at P11 and P21 had a significant but transient effect on the Morris water maze spatial learning, since additional hyperthermia episode failed to induce cognitive deficit and Tr3 rats became indistinguishable from corresponding controls. These findings correlated with those of Notenboom et al. [33] who, throughout electrophysiological and histochemical study, found enhanced hippocampal CA1 long-term potentiation and reduced long-term depression without alteration of spatial learning and memory in Morris water maze in adult rats (P60) that were subjected to experimental febrile seizures at P10, due to the modulation of synaptic plasticity in hippocampus. Our results are also in agreement with the findings of Dubé et al. [34] reporting in P90-old rats moderate deficits in working and reference memory in Morris water maze after early-life febrile seizures. On the other hand, our observations are in disagreement with those of Chang et al. [35] who found long-term memory impairment at adulthood following febrile seizures early in life.

In the probe test, Tr1 and Tr2 rats spent also less significant time in the target quadrant than corresponding controls, whereas Tr3 and C3 groups spent the same time in this quadrant. Moreover, with age, control and treated groups tended to spend more time in the target quadrant, but the effect was significant only for C3 and Tr3. Indeed, hyperthermia-induced seizures early in life may impair spatial memory consolidation. These observations could be correlated with studies showing that rats with hippocampus, dentate gyrus, subiculum, or combined damage did not perform well in tests without a platform [36, 37]. Similarly, in a genetic model of epilepsy displaying spontaneous spike-and-wave discharges, behavioural analysis using Morris water maze indicated learning deficit compared to wild type littermates [38].

Memory transfer test showed that control rats were generally more efficient in the transfer of learning and memorizing the new position of the visible platform (southwest quadrant) than corresponding treated groups, but the differences were only significant for C3 group. These observations indicated that repetitive febrile seizures might affect the ability to memorize and use spatial information to learn the novel location of the platform.

No sex differences were observed in learning and visuospatial memory in the Morris water maze [39], that in all our experiments male and female rats were pooled in the same group.

Taken together, our results showed that febrile seizures in low age affected mainly spatial acquisition of cognitive abilities and the alterations seemed to disappear later, confirming in part previous behavioural animal studies demonstrating that early-life prolonged febrile seizures resulted in moderate or absence of alteration of cognitive performance at adulthood [33, 34]. Nevertheless, cognitive defects were not excluded after the testing age we used.

It has been shown that duration of febrile seizures influenced the probability of developing subsequent epilepsy and severity of the spontaneous seizures. Thus, febrile status averaging 64 min was more efficient compared to 24 min to increase the severity and the duration of febrile seizures [13]. Therefore, the moderate impact of febrile seizures registered in our study could be explained in part by the restricted duration of treatment (30 min).

## 5. Conclusion

With a novel methodology using hyperthermia hot water, we showed that the possible hippocampus damage being caused by repetitive hyperthermia-induced seizures in immature rats might be moderate and transient. Thus, further investigations using especially biochemical and histopathological markers of hippocampal alteration are required to better characterize this model.

## Figures and Tables

**Table 1:** Generalised tonic-clonic seizure appearance time (GTCSAT) in treated group 1 (Tr1, *n* = 15), treated group 2 (Tr2, *n* = 10), and treated group 3 (Tr3, *n* = 7). ^∗^ *P* < 0.05 versus Tr1.

Group	Tr1	Tr2	Tr3
Time (min)	16.06 ± 0.60	13.33 ± 0.51^∗^	12.46 ± 0.39^∗^

**Table 2:** Average latency to reach the hidden platform from the north and west starting points for control (C1, *n* = 18), (C2, *n* = 10), and (C3, *n* = 7) and treated (Tr1, *n* = 18), (Tr2, *n* = 10), and (Tr3, *n* = 11) rats by the 4th learning day in the Morris water maze. Data are shown as mean values ± SEM. Values not sharing the same letter are significantly different; *P* < 0.01.

	Group	North	West
Time (sec)	C1	19.57 ± 3.22^a^	17.85 ± 3.38^a^
	Tr1	38.75 ± 5.04^b^	30.23 ± 3.88^b^
	C2	20.50 ± 5.33^a^	14.50 ± 1.38^a^
	Tr2	44.12 ± 5.75^b^	36.80 ± 7.35^b^
	C3	7.50 ± 1.70^c^	4.30 ± 0.65^c^
	Tr3	5.54 ± 1.03^c^	7.72 ± 2.37^c^

**Table 3:** Average time spent by control (C1, C2, and C3) and treated (Tr1, Tr2, and Tr3) groups in target quadrant. Data are shown as mean values ± SEM. Values not sharing the same letter are significantly different; *P* < 0.01.

Group	C1	Tr1	C2	Tr2	C3	Tr3
Time (sec)	22.31 ± 1.90^a^	17.13 ± 2.20^b^	28.18 ± 4.93^a^	19.07 ± 3.10^b^	51.44 ± 5.94^c^	47.53 ± 5.60^c^

**Table 4:** Average latency to reach the visible platform (southwest quadrant) for control (C1, C2, and C3) and treated (Tr1, Tr2, and Tr3) groups after north or west introduction. Data are shown as mean values ± SEM. ^∗^ *P* < 0.05 versus corresponding control.

	Group	North	West
Time (sec)	C1	24.16 ± 7.08	13.21 ± 6.92
	Tr1	25.61 ± 7.49	18.13 ± 7.90
	C2	25.60 ± 3.98	12.60 ± 3.84
	Tr2	29.01 ± 5.99	18.30 ± 5.49
	C3	23.45 ± 2.44	13.18 ± 2.95
	Tr3	34.09 ± 3.04^∗^	25.81 ± 6.55^∗^
